# Supplementary material for: Endogenous formaldehyde scavenges cellular glutathione resulting in redox disruption and cytotoxicity
Source: Nat Commun. 2022 Feb 8;13:745. doi: 10.1038/s41467-022-28242-7 (PMC8827065; doi:10.1038/s41467-022-28242-7)
Supplement: Supplementary file 1 — Supplementary Information [file 41467_2022_28242_MOESM1_ESM.pdf]

Endogenous formaldehyde scavenges cellular glutathione resulting in redox disruption and cytotoxicity

Carla Umansky[1,6], Agustín E. Morellato[1,6], Matthias Rieckher[2,6], Marco A. Scheidegger[1], Manuela R. Martinefski[3], Gabriela A. Fernandez[3], Oleg Pak[4], Ksenia Kolesnikova[2], Hernán Reingruber[1], Mariela Bollini[3], Gerry P. Crossan[5], Natascha Sommer[4], María Eugenia Monge[3], Björn Schumacher[2] and Lucas B. Pontel[1,*]

Supplementary Fig. 1

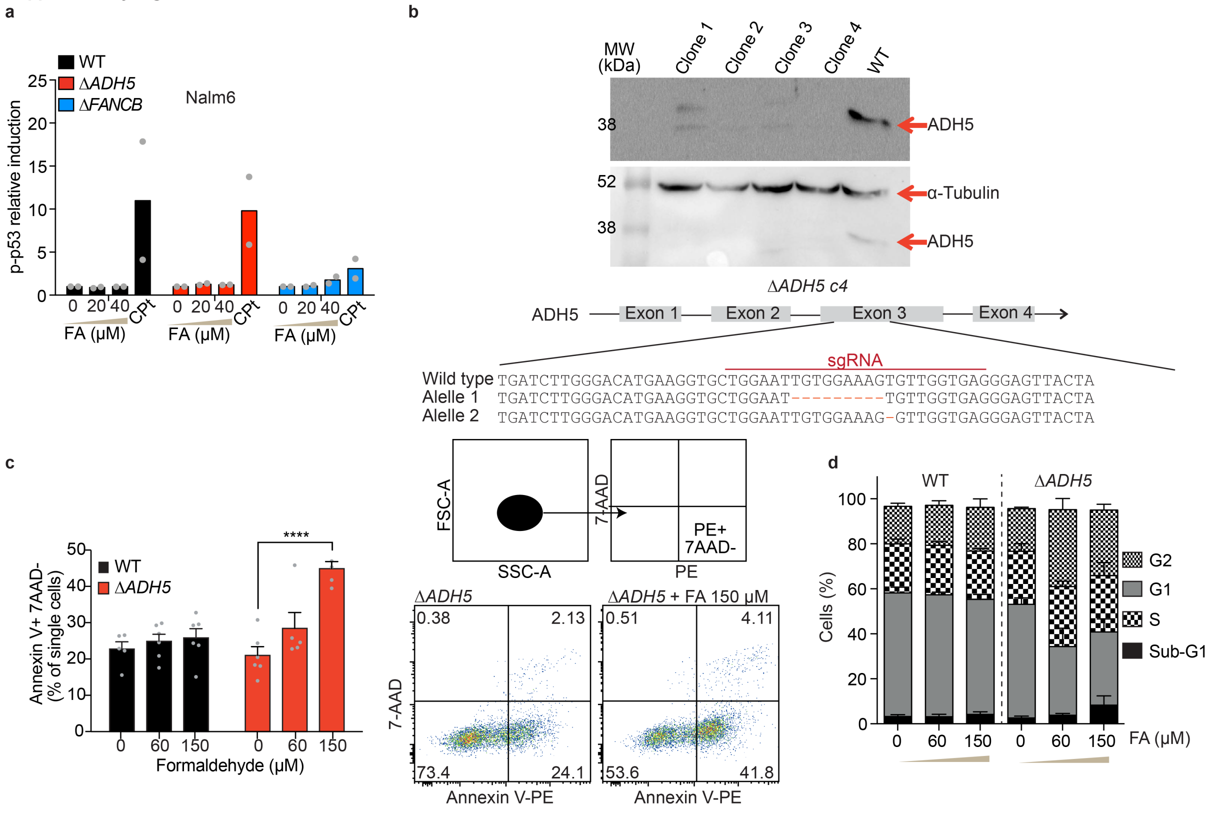

Supplementary Figure 1. **Formaldehyde is cytotoxic for Δ*ADH5* cells. a** Quantification of p-p53 western blots shown in Fig. 1b using ImageJ (n=2 biological independent samples, mean comparing against the same cell line untreated). WT: Wild type, FA: Formaldehyde, CPt: Cisplatin. µM refers to µmol L^{-1}. **b** Western blot analysis of ADH5 expression in clones edited by CRISPR/Cas9 in HCT116 (Top: ADH5; Bottom: same membrane stripped and blotted against α-Tubulin). Bottom: *ADH5* gene showing the exon targeted by CRISPR/Cas9 and the genetic modifications of the HCT116 Δ*ADH5* clone used in this work. **c** Quantification of apoptosis determined by Annexin V detection in WT and Δ*ADH5* HCT116 cells exposed to FA over 24 h at the concentrations indicated in the figure (n=5 biological independent samples, mean ± SEM, two-tailed unpaired *t*-test, *****P*<0.0001). Right: Representative flow cytometry plot for phosphatidylserine determination by Annexin V in Δ*ADH5* HCT116 cells untreated or exposed to 150 µmol L^{-1} FA (7-AAD: 7-amino-actinomycin D) and gating strategy (Top). **d** Cell cycle analysis of WT and Δ*ADH5* HCT116 cells exposed to 60 and 150 µmol L^{-1} FA over 24 h (n=4, mean ± SEM).

Suplementary Fig. 2

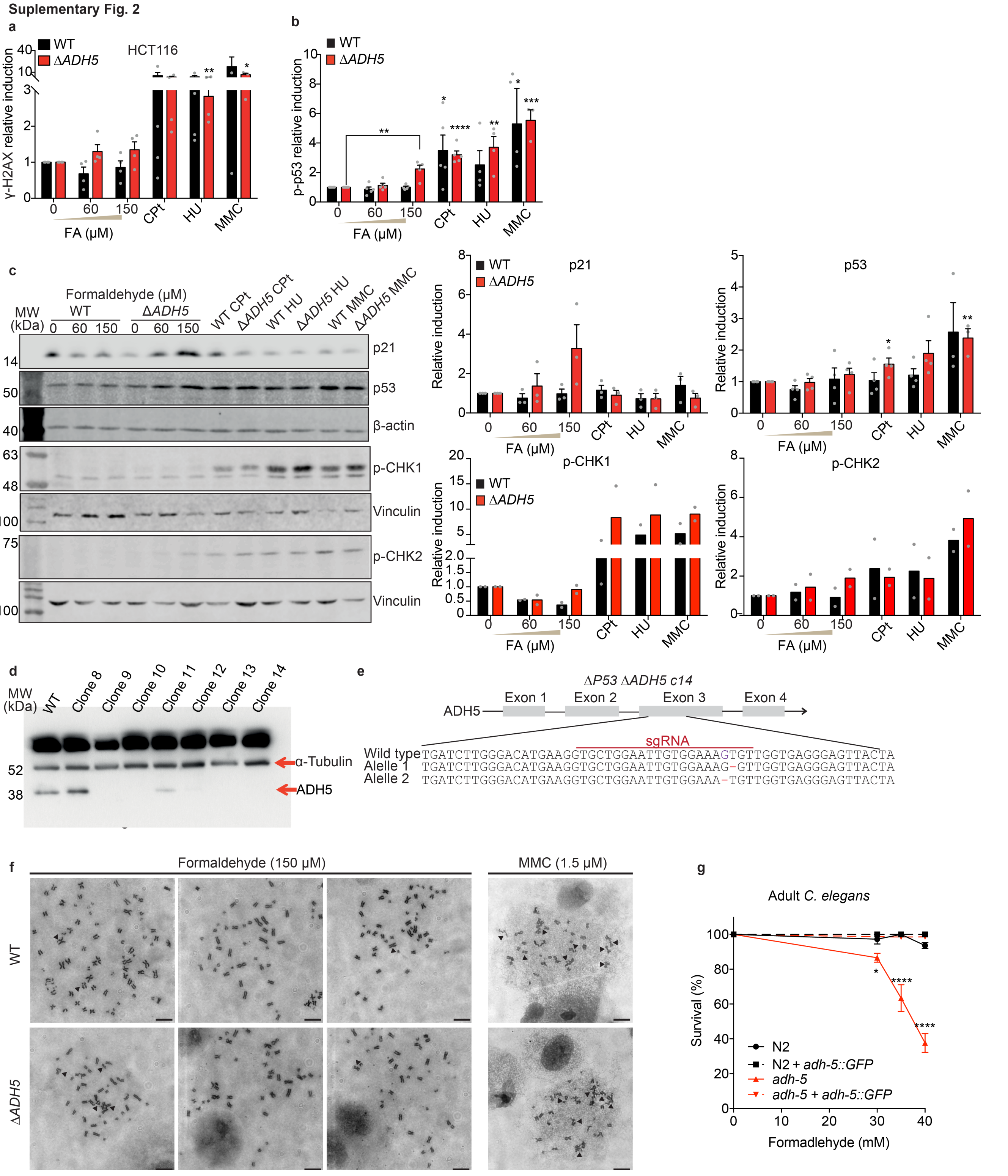

Supplementary Figure 2. ***ΔADH5 ΔTP53* HCT116 cell line generation and DNA damage. a** Quantification of γ-H2AX western blots shown in fig. 1f (Formaldehyde (FA) 0, 60, 150, Cisplatin (CPt), and Hydroxyurea (HU) n=4; Mitomycin C (MMC) n=3; mean ± SEM, two-tailed unpaired *t*-test comparing against same cell line untreated, ***P*=0.0025 (Δ*ADH5*, HU), **P*=0.021(Δ*ADH5,* MMC)). WT: Wild type. **b** Quantification of p-p53 western blots shown in fig. 1f (FA 0, 60, 150 and CPt n=5; HU n=4, MMC n=3, two-tailed unpaired *t*-test comparing against same cell line untreated, ***P*=0.0014 (Δ*ADH5* vs Δ*ADH5* 150 μmol L^{-1} FA), **P*=0.045 (WT, Cpt), ***P*=0.0038 (Δ*ADH5*, HU), ****P*=0.0001 (Δ*ADH5,* MMC), **P*=0.0496 (WT, MMC), *****P*<0.0001)). **c** Western blots against

p21, p53, p-CHK1 and p-CHK2 with the corresponding loading controls in WT and Δ*ADH5* HCT116 cells. p-CHK1 and the corresponding vinculin control were run on different gels. Uncropped images in Source Data File. Right: Quantification of western blots against p21 (n=3, mean ± SEM, two-tailed unpaired *t*-test comparing against same cell line untreated) and p53 (n=4 all but MMC with n=3, mean ± SEM, two-tailed unpaired *t*-test comparing against same cell line untreated, **P*=0.0298 ***P*=0.0024); and p-CHK1 and p-CHK2 (mean, n=2). **d** Western blot analysis of ADH5 expression in HCT116 Δ*TP53* clones edited by CRISPR/Cas9. **e** *ADH5* gene showing the exon targeted and the genetic modifications of the HCT116 Δ*TP53* Δ*ADH5* clone used in this work. **f** Representative images of metaphases generated with WT and Δ*ADH5* HCT116 cells exposed to 150 µmol L^{-1} FA or 1.5 µmol L^{-1} MMC (scale bar 1 µm). **g** Survival of adult *C. elegans* expressing the transgene *adh-5::GFP* exposed to the indicated concentrations of FA for 4 h (n=3, mean ± SEM, two-way ANOVA Bonferroni's multiple comparisons test, **P*=0.0232, *****P*<0.0001 (*adh-5* vs N2, same FA concentration)).

Supplementary Fig. 3

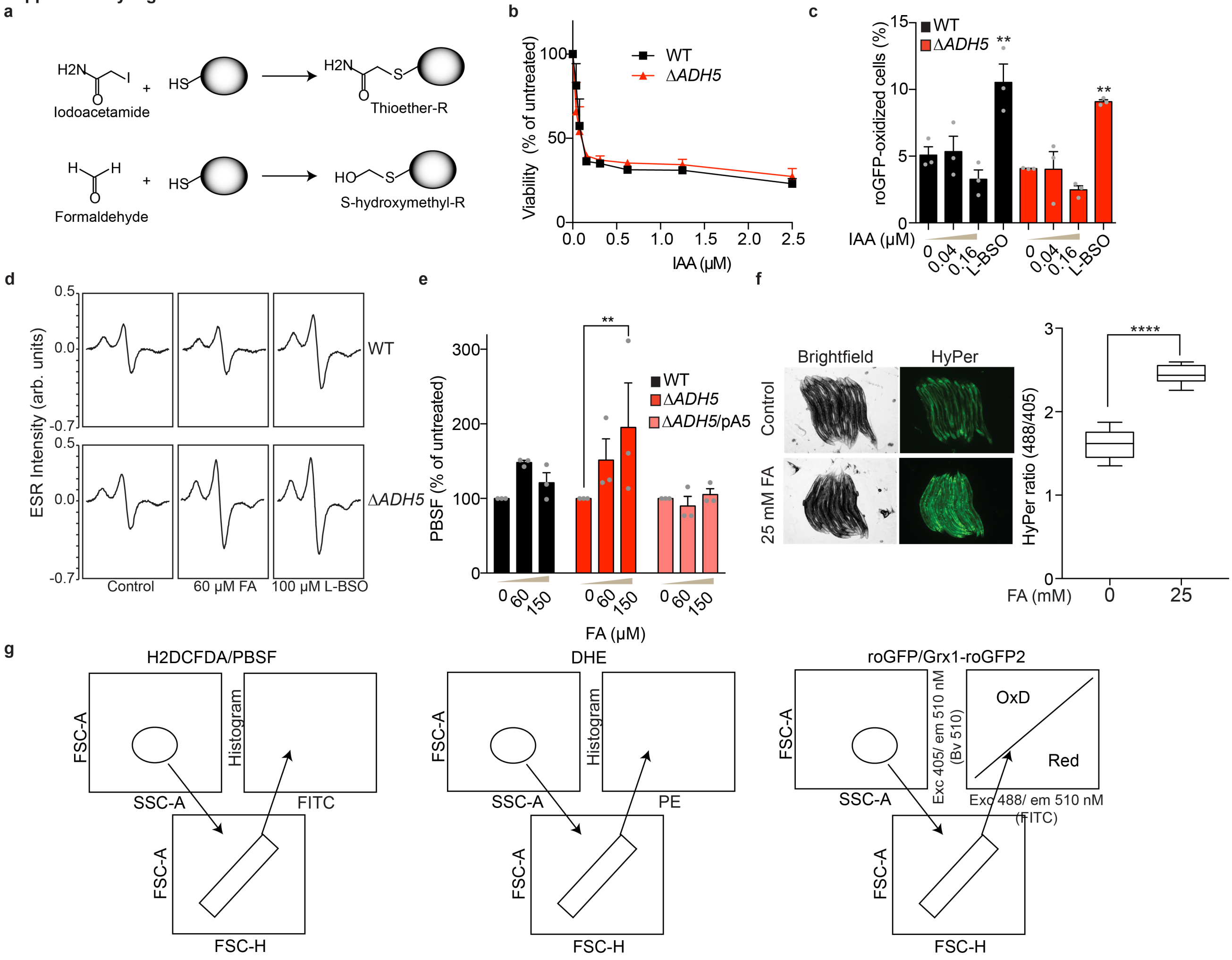

Supplementary Fig. 3. **Formaldehyde induces oxidative stress. a** Comparison of the reaction of iodoacetamide (IAA) with thiol groups (top) with the reaction of formaldehyde (FA) with thiol groups (bottom). **b** Viability assay in Wild type (WT) and Δ*ADH5* HCT116 cells in presence of different concentrations of IAA. Viability was determined after five days relative to the corresponding untreated samples (n=3, mean ± SEM). µM refers to µmol L^{-1}. **c** Plot depicting the percentage of WT and Δ*ADH5* HCT116 cells harbouring the cytoplasmic-roGFP reporter that show oxidation after exposure to 0.04 and 0.16 µM IAA for 48 h. L-buthionine sulfoximine (L-BSO) is shown as positive control (n=3, mean ± SEM, two-way ANOVA corrected for Tukey's multiple

comparison **P=0.0046 (WT), **P=0.0086 (Δ*ADH5*)). **d** Representative plots obtained from Electron Spin Resonance (ESR) spectroscopy in WT and Δ*ADH5* HCT116 cells exposed to 60 µmol L^{-1} FA or to 100 µmol L^{-1} L-BSO for 48 h. **e** Cellular Peroxides determination by pentafluorobenzenesulfonyl fluorescein (PBSF) in Wild type (WT), Δ*ADH5*, and complemented Δ*ADH5* (Δ*ADH5/*pA5) HCT116 cells upon 48 h exposure to 0, 60 and 150 µmol L^{-1} FA. Data is represented as the % of untreated from the same cell line (n=3, mean ± SEM, two-way ANOVA, Tukey´s multiple comparison test, **P=0.008). mM refers to mmol L^{-1}. **f** Left, representative images of adult *C. elegans* expressing the H2O2 sensor HyPer exposed to 0 or 25 mmol L^{-1} FA for 5 h. Right, quantification of Hyper ratiometric response after excitation at 488 nm and 405 nm, denoting the induction of HyPer by 25 mmol L^{-1} FA (n=10, box (line at median) and whiskers (min and max), ****P<0.0001, unpaired non-parametric two-tailed Mann-Whitney test). **g** FACS gating approaches for H2DCFDA (Fig. 3b), PBSF (Sup. Fig 3e), DHE (Fig. 3c), roGFP (Fig. 3e) and Grx1-roGFP2 (Fig. 6i).

Supplementary Fig. 4

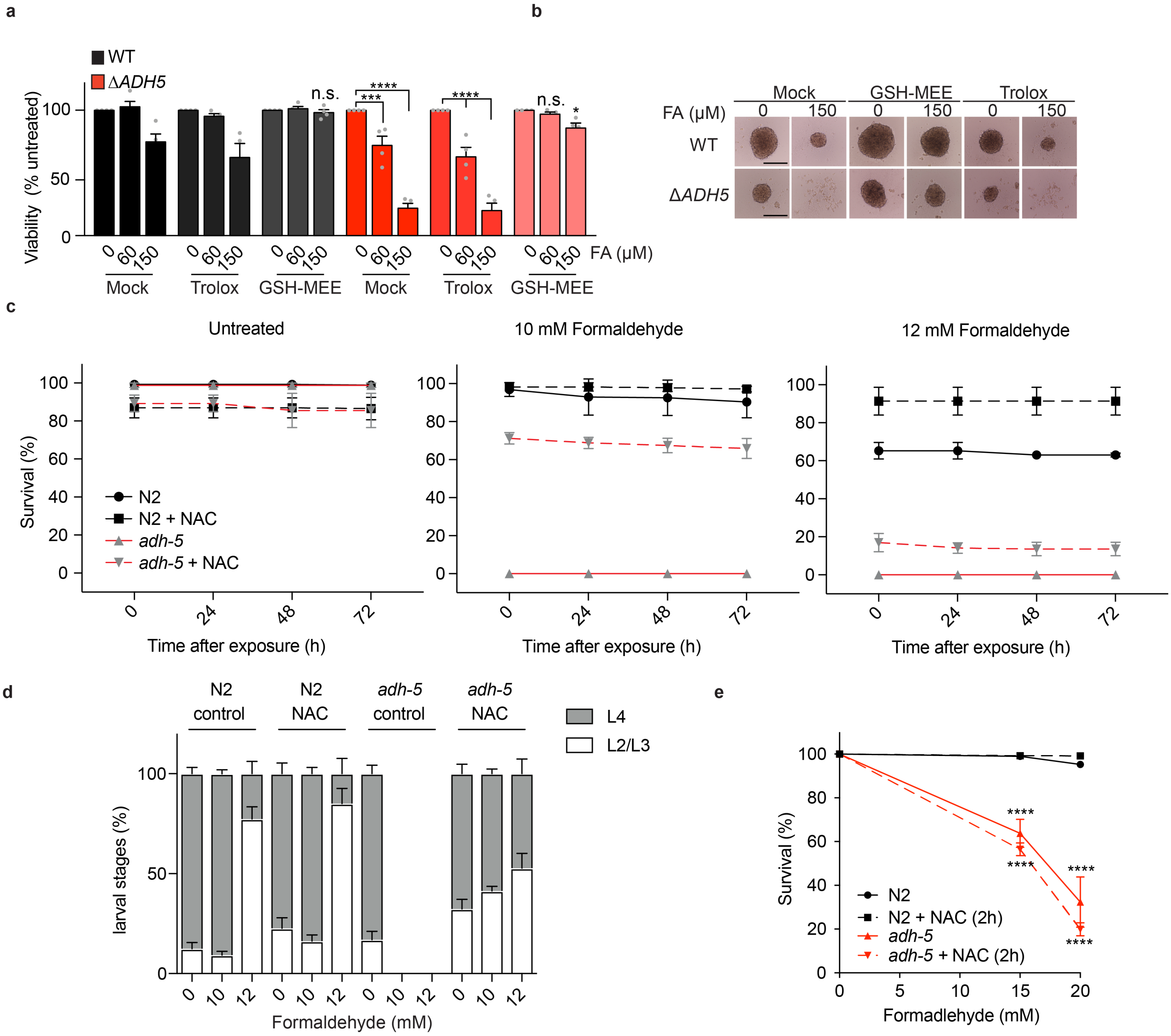

Supplementary Figure 4. **Formaldehyde cytotoxicity is prevented by thiol-containing antioxidants. a** Viability assays in presence of 60 or 150 µmol L^{-1} formaldehyde (FA) and Trolox (1 mmol L^{-1}) or glutathione monoethyl ester (GSH-MEE, 1 mmol L^{-1}). L-BSO was used at 100 µmol L^{-1} in Wild type (WT) and Δ*ADH5* HCT116 cells (n=4, mean ± SEM; two-way ANOVA, Tukey multiple comparison test, ***P=0.0002,****P<0.0001, *P=0.03, n.s. not

significative against same cell line untreated. μM refers to μmol L^{-1} and mM refers to mmol L^{-1}. **b** Extended data for the experiment shown in Fig. 4b. Representative images of sphere-formation assay carried out in presence of 150 μmol L^{-1} FA and Trolox (1 mmol L^{-1}) or GSH-MEE (1 mmol L^{-1}). As reference, the pictures labelled as mock in this panel are the same as the ones included in the main Fig. 4b. **c** Survival of L1-staged Wild type (N2) and *adh-5 C. elegans* mutant upon exposure to the indicated FA concentrations and 10 mmol L^{-1} N-acetyl-cysteine (NAC) measured 0, 24, 48 and 72 h after treatment (n=3, mean ± SD). **d** Developmental stages of surviving animals 48 h after FA exposure (n=3, mean ± SD). **e** Survival of adult N2 and *adh-5 C. elegans* mutant pre-exposed for 2 h to 10 mmol L^{-1} NAC, washed off and then exposed to the indicated FA concentrations for 4 h (n=3, mean ± SD, two-way ANOVA with Tukey comparison test against N2 same FA concentration ****$P<0.0001$). Survival was scored right after the FA treatment.

Supplementary Fig. 5

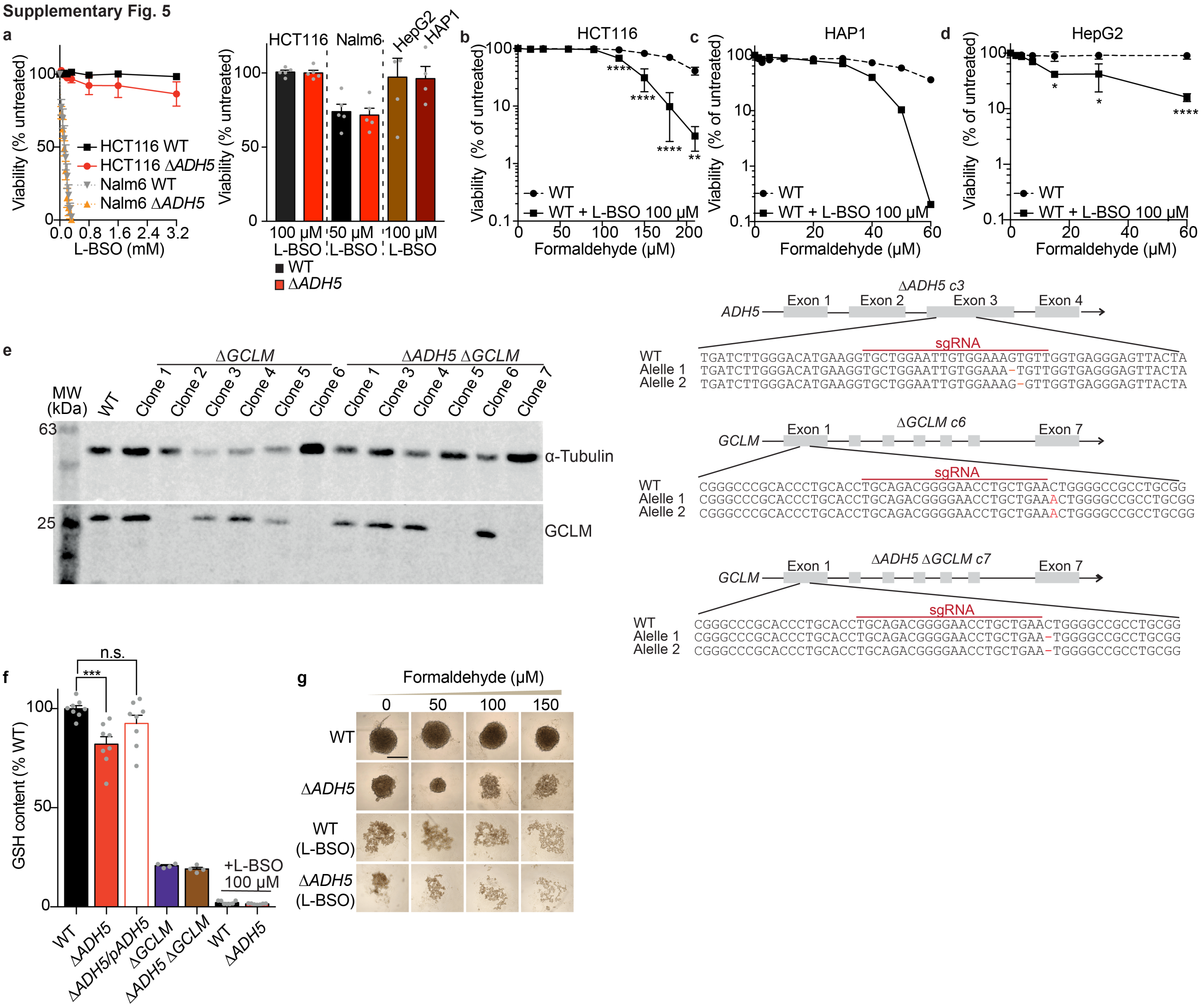

Supplementary Figure 5. **GSH biosynthesis inactivation. a** Viability assay for Wild type (WT) and Δ*ADH5* HCT116 and Nalm6 cells in response to increasing concentrations of L-buthionine-sulfoximine (L-BSO) determined after three (HCT116, n=6 (WT), n=3 (Δ*ADH5*), mean ± SEM) or five (Nalm6, n=6, mean ± SEM) days. μM refers to μmol L^{-1}. Right: Viability assay for HCT116 and Nalm6 WT and Δ*ADH5* cells at 100 and 50 μmol L^{-1} L-BSO, respectively (n=5, mean ± SEM); HepG2 (n=5, mean ± SEM) and HAP1 (n=4, mean ± SEM) cell lines at 100 μmol L^{-1} L-BSO. **b** Formaldehyde (FA) dose-response viability assay for HCT116 cells in presence of 100 μmol L^{-1} L-BSO (n=5, mean, two-way ANOVA corrected for Bonferroni's multiple comparisons test

****P<0.0001, *P=0.0033). **c** Formaldehyde (FA) dose-response viability assay for HAP1 cells in presence of 100 µmol L^{-1} L-BSO (n=2, mean). **d** FA dose-response MTS viability assay for HepG2 cells in presence of 100 µmol L^{-1} L-BSO (n=3, mean ± SEM, two-way ANOVA corrected for Bonferroni's multiple comparisons test *P=0.0182 (15 µM FA), *P=0.011 (30 µM FA), ****P<0.0001,). **e** Western blot analysis of *GCLM* expression in HCT116 clones edited by CRISPR/Cas9. Right: *ADH5* and *GCLM* genes showing the exon targeted by CRISPR/Cas9 and the genetic modifications detected. Bottom: *GCLM* gene showing the exon targeted by CRISPR/Cas9 and the genetic modifications of the HCT116 Δ*GCLM* clones used in this work. **f** Bar plot for GSH content in WT (n=8), Δ*ADH5* (n=8), Δ*ADH5/*pADH5 (n=8), Δ*GCLM* (n=4) and Δ*ADH5* Δ*GCLM* (n=4) cells, and in WT (n=7) and Δ*ADH5* (n=7) HCT116 cells exposed to 100 µmol L^{-1} L-BSO for 48 h. Every dot is the percentage of fluorescence intensity in a single well relative to the average fluorescence of WT samples run the same day and corrected for viability determined using resazurin (mean ± SEM, one-way ANOVA with Tukey test for multiple comparisons,***P=0.0001). **g** 3D-spheroid formation in presence of 100 µmol L^{-1} L-BSO and the indicated concentrations of FA in HCT116 cells. Pictures were taken 5 days after seeding cells on agarose-coated plates (scale bar 0.5 mm).

Supplementary Fig. 6

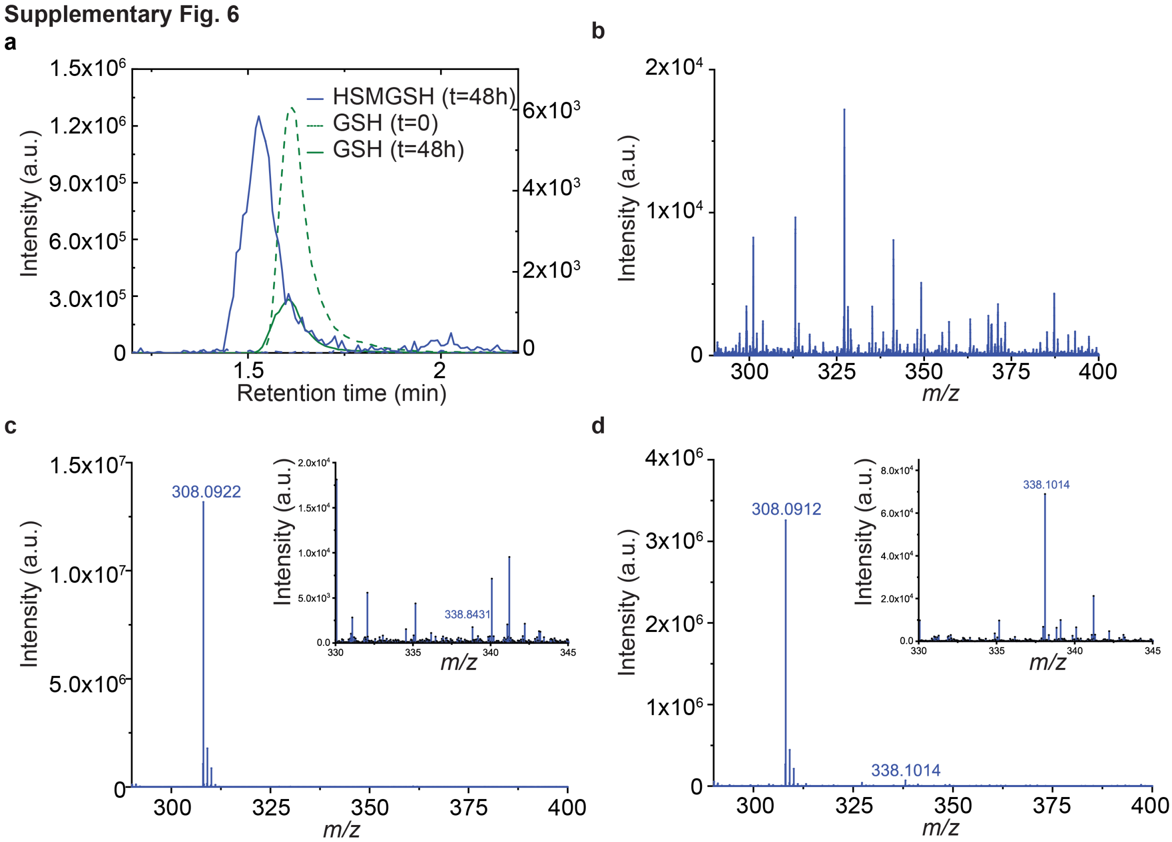

Supplementary Figure 6. **S-hydroxymethylglutathione synthesis.** **a** Extracted ion chromatograms for [glutathione (GSH) + H$]^+$ ion at *m/z* 308.0916 generated from a 10.4 µmol L^{-1} GSH standard solution before reaction (t_0: green dash line) and after 48 h of reaction (t_{48}: green solid line); and for [S-hydroxymethylglutathione (HSMGSH) + H$]^+$ ion at m/z 338.1022 generated from a 10.4 µmol L^{-1} GSH standard solution before reaction (t_0: blue dash line) and after 48 h reaction (t_{48}: blue solid line). **b** Mass spectrum for the solvent at t_0, with no signals detected at *m/z* 308.0916 or *m/z* 338.1022. **c** Mass spectrum for a GSH standard solution at t_0, with no signal detected at *m/z* 338.1022. **d** Mass spectrum for the reaction mixture at t_{48}.

Supplementary Fig. 7

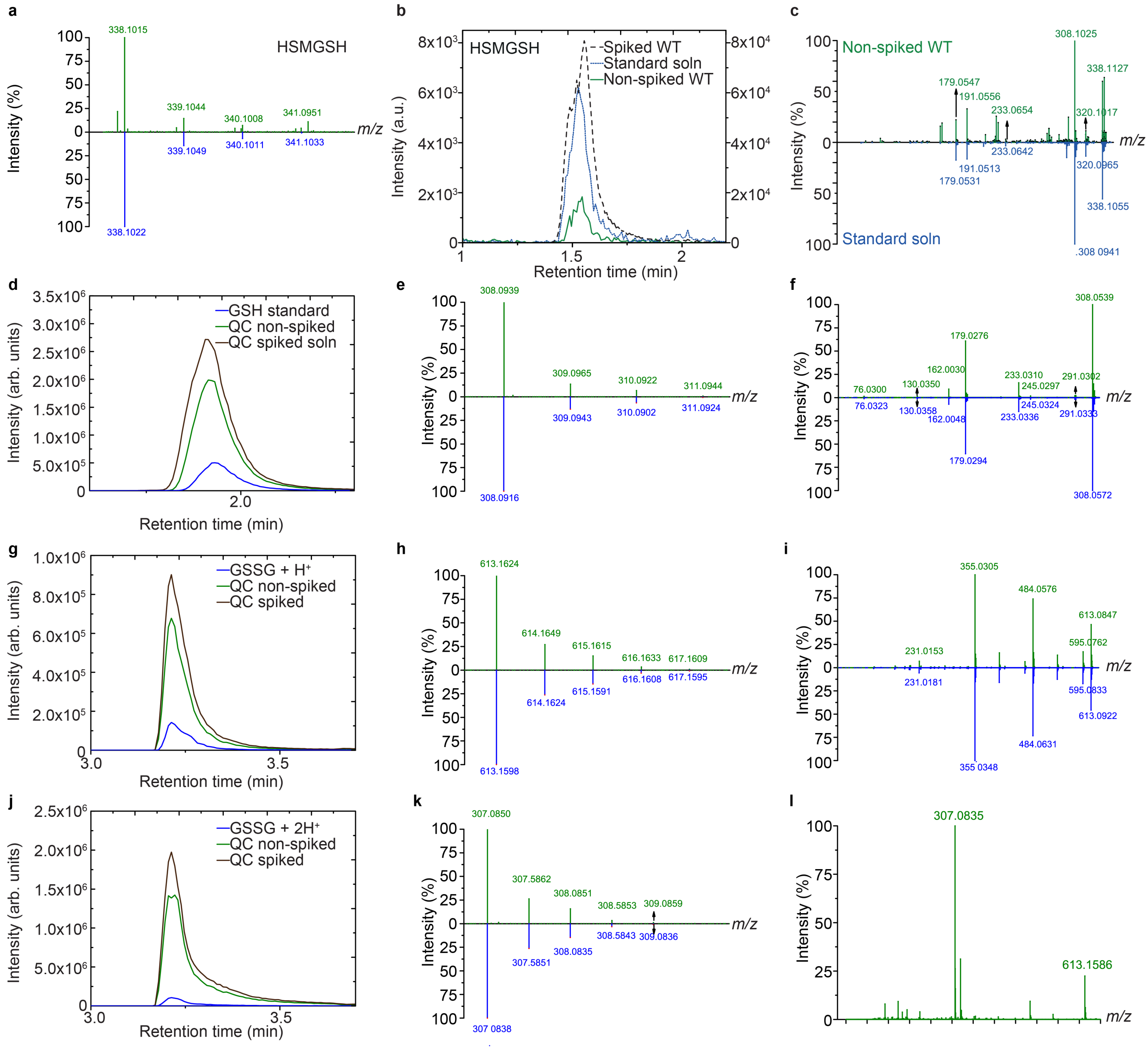

Supplementary Fig. 7: detection of S-hydroxymethylglutathione in cells.

a. Mass spectrum for [S-hydroxymethylglutathione (HSMGSH) + H]$^+$ ion at *m/z* 338.1022 in a Wild type (WT) sample (green), and its simulated isotopic pattern (blue). **b.** Extracted ion chromatograms for [HSMGSH + H]$^+$ ion at m/z 338.1022 ± 0.0500. Non-spiked WT sample (green, left axis), 20 µmol L^{-1} spiked WT (black dotted line, right axis), and 20 µmol L^{-1} HSMGSH standard solution (blue, left axis)**.** **c.** Product ion mass spectra of [HSMGSH + H]$^+$ precursor ion. WT (green) and a 20 µmol L^{-1} HSMGSH standard solution (blue), using a collision cell voltage of 10 V. **d.** Extracted ion chromatograms for [GSH + H]$^+$ ion at *m/z* 308.0916 ± 0.0500. Non-spiked QC sample (green), 43 µmol L^{-1} spiked QC sample (brown), and 14.3 µmol L^{-1} GSH standard solution (blue). **e.** Mass spectrum for [GSH + H]$^+$ ion at *m/z* 308.0916. QC sample (green), and its simulated isotopic pattern (blue). **f.** Product ion mass spectrum for [GSH + H]$^+$ precursor ion. QC sample (green) and 14.3 µmol L^{-1} GSH standard solution (blue), using a collision cell voltage of 10 V. **g.** Extracted ion chromatograms for [GSH disulfide (GSSG) + H]$^+$ ion at *m/z* 613.1598 ± 0.0500. Non-spiked QC sample (green), 15.5 µmol L^{-1} spiked QC sample (brown), and 15.5 µmol L^{-1} GSSG standard (blue). **h.** Mass spectrum for [GSSG + H]$^+$ ion at *m/z*

613.1598. QC sample (green), and its simulated isotopic pattern (blue). **i.** Product ion mass spectrum for $[GSSG + H]^+$ precursor ion. QC sample (green), and a 15.5 µmol L^{-1} GSSG standard solution (blue) using a collision cell voltage of 20 V.
