# Supplementary material for: Endogenous formaldehyde scavenges cellular glutathione resulting in redox disruption and cytotoxicity
Source: Nat Commun. 2022 Feb 8;13:745. doi: 10.1038/s41467-022-28242-7 (PMC8827065; doi:10.1038/s41467-022-28242-7)
Supplement: Supplementary file 3 — Reporting Summary [file 41467_2022_28242_MOESM3_ESM.pdf]

nature portfolio

Corresponding author(s): Lucas B. Pontel

Last updated by author(s): Dec 10, 2021

Reporting Summary

Nature Portfolio wishes to improve the reproducibility of the work that we publish. This form provides structure for consistency and transparency in reporting. For further information on Nature Portfolio policies, see our Editorial Policies and the Editorial Policy Checklist.

Statistics

For all statistical analyses, confirm that the following items are present in the figure legend, table legend, main text, or Methods section.

n/a	Confirmed	
[ ]	[x]	The exact sample size (*n*) for each experimental group/condition, given as a discrete number and unit of measurement
[ ]	[x]	A statement on whether measurements were taken from distinct samples or whether the same sample was measured repeatedly
[ ]	[x]	The statistical test(s) used AND whether they are one- or two-sided<br>*Only common tests should be described solely by name; describe more complex techniques in the Methods section.*
[ ]	[x]	A description of all covariates tested
[ ]	[x]	A description of any assumptions or corrections, such as tests of normality and adjustment for multiple comparisons
[ ]	[x]	A full description of the statistical parameters including central tendency (e.g. means) or other basic estimates (e.g. regression coefficient) AND variation (e.g. standard deviation) or associated estimates of uncertainty (e.g. confidence intervals)
[ ]	[x]	For null hypothesis testing, the test statistic (e.g. *F*, *t*, *r*) with confidence intervals, effect sizes, degrees of freedom and *P* value noted<br>*Give P values as exact values whenever suitable.*
[x]	[ ]	For Bayesian analysis, information on the choice of priors and Markov chain Monte Carlo settings
[x]	[ ]	For hierarchical and complex designs, identification of the appropriate level for tests and full reporting of outcomes
[x]	[ ]	Estimates of effect sizes (e.g. Cohen's *d*, Pearson's *r*), indicating how they were calculated

Our web collection on statistics for biologists contains articles on many of the points above.

Software and code

Policy information about availability of computer code

Data collection	BD FACSDiva TM software v6.1<br>MassLynx version 4.1 (Waters Corp., Milford, MA, USA).
Data analysis	Sofware used for data analysis:<br>ImageJ version 2.0.0-rc-69/1.52p.<br>GraphPad v7.0a<br>TreeDyn 193.8 (phylogeny.fr) for phylogenetic analysis.<br>FlowJo 10.0.7 (Tree Star)

For manuscripts utilizing custom algorithms or software that are central to the research but not yet described in published literature, software must be made available to editors and reviewers. We strongly encourage code deposition in a community repository (e.g. GitHub). See the Nature Portfolio guidelines for submitting code & software for further information.

Data

Policy information about availability of data

All manuscripts must include a data availability statement. This statement should provide the following information, where applicable:

- Accession codes, unique identifiers, or web links for publicly available datasets
- A description of any restrictions on data availability
- For clinical datasets or third party data, please ensure that the statement adheres to our policy

1. Sequences of human ADH5 ortholog genes were obtained from NCBI (by perfoming a BlastP against human ADH5 and downloading the fasta file with the output. The sequences used are listed in the Source Data File.

2. Source Data for figures are provided in the accompanying "Source Data File".

Field-specific reporting

Please select the one below that is the best fit for your research. If you are not sure, read the appropriate sections before making your selection.

- [x] Life sciences
- [ ] Behavioural & social sciences
- [ ] Ecological, evolutionary & environmental sciences

For a reference copy of the document with all sections, see nature.com/documents/nr-reporting-summary-flat.pdf

Life sciences study design

All studies must disclose on these points even when the disclosure is negative.

Sample size	The sample size was estimated based on the experience and according to results from pilot experiments. For viability/survival curves, when a clear phenotype was observed in the pilot experiment, a minimum of 3 replicates were performed. For Mass spectrometry and ESR we arbitrary performed a minimum of 5 biological replicates, based on previous expertise. For ESR, the experiments were set up to obtain 6 independent measurements of each condition/cell line (total of 72 samples). After processing, 4 samples were below the minimum number of cells (2x106) required and were not run in the EPR machine.
Data exclusions	Principal component analysis (PCA) was conducted using MATLAB R2015a (The MathWorks, Natick, MA, USA) with the PLS Toolbox version 8.1 (Eigenvector Research, Inc., Manson, WA, USA). PCA was used to track data quality and to identify and remove outliers in the dataset. Two samples were identified as outliers by PCA, one from WT and one from ΔADH5 cells, and were not further considered for data analysis. For experiment in Fig. 7a strain ΔFANCI, one of the replicates was contaminated and only 2 biological independent replicates are available, which present similar profiles.
Replication	Multiple independent repeats were included for related experiments and shown as dots in figures. Each experiment was performed for at least twice to corroborate data are reproducible.
Randomization	The allocation of samples in this study was performed according to their genetic background (usually WT vs knockout). Then, the allocated samples were exposed to the experimental conditions simultaneously, and results collected at the indicated end point. For example, for a viability assay, technical triplicates of both groups were contained in the same plate, exposed to the drug(s) and revealed simultaneously by the selected readout method. The same process was applied to all the experiments comparing different genetic backgrounds and different conditions. For UPLC-HRMS experiments, samples were generated according to the allocation protocol described above. Frozen extracts were labeled as a1, b1, c1, etc. The samples were randomly analyzed within a defined template of spiked QC samples, and the analysis order was balanced based on sample classes. QC samples were used to condition the LC-MS system before sample analysis.
Blinding	Blinding was used for collection and scoring of metaphases and imaging of C. elegans (gst4-GFP induction). Researcher (R1) generated slides (or samples for UPLC-HRMS) and labelled them as A1, A2, A3, etc. R1 was not blinded, and provided the anonymized slides to a microscopy technician, who took images assigning a name to every image (SlideA1_1, SlideA1_2, etc). The images were provided to a researcher (R2) that scored every image into an excel file. (SlideA1_1 X abnormalities, SlideA1_2 no abnormalities, etc). The excel file was provided to R1, who assigned the id of each slide and plot the data.<br>Blinding was not relevant on viability/survival and ROS detection experiments because the readout (FACS data collection, viability readout, etc) and the calculations were performed by a computer.

Reporting for specific materials, systems and methods

We require information from authors about some types of materials, experimental systems and methods used in many studies. Here, indicate whether each material, system or method listed is relevant to your study. If you are not sure if a list item applies to your research, read the appropriate section before selecting a response.

Materials & experimental systems

n/a	Involved in the study	
[ ]	[x]	Antibodies
[ ]	[x]	Eukaryotic cell lines
[x]	[ ]	Palaeontology and archaeology
[ ]	[x]	Animals and other organisms
[x]	[ ]	Human research participants
[x]	[ ]	Clinical data
[x]	[ ]	Dual use research of concern

Methods

n/a	Involved in the study	
[x]	[ ]	ChIP-seq
[ ]	[x]	Flow cytometry
[x]	[ ]	MRI-based neuroimaging

Antibodies

Antibodies used	Antibodies from Cell Signaling Technology: p53 Cat#9282/RRID: AB_331476; p-p53 Cat#9284/RRID: AB_331464; p21 Cat#2947/ RRID:AB_823586; phospho-CHK1 Cat#2348/RRID:AB_331212; phospho-CHK2 Cat# 2661/RRID:AB_331479; phospho-histone H2A.X Cat#9718/RRID: AB_2118009; alpha-Tubulin Cat#2144/RRID:AB_2210548; HRP-linked anti-rabbit Cat#7074/RRID:AB_2099233; HRP-

linked anti-mouse Cat#7076/RRID:AB_330924; antiDyLight-800 4x PEG-linked anti-rabbit Cat#5151;DyLight-680-linked anti-mouse Cat#5470. Antibodies from Santa Cruz Biotechnology: Vinculin, Cat#sc-73614/ RRID: AB_1131294; beta-actin Cat#sc-47778/ RRID:AB_626632. Antibodies from Atlas Antibodies: GCLM Cat# HPA023696/RRID:AB_1849560. Anti-DDDDK tag (FLAG) from Abcam (ab49763/RRID:AB_869428). Antibody against ADH5 was obtained from KJ Patel laboratory (Pontel, et al 2015). Anti-Annexin V antibody was from BD (BD Biosciences Cat# 559763, RRID:AB_2869265)

Validation

Antibodies from Cell Signaling Technology:

p53 Cat#9282/RRID: AB_331476
Antibody was validated against human by the manufacturer, stating “p53 Antibody recognizes endogenous levels of total p53 protein. This antibody binding has been mapped to the amino terminus and DNA binding domain of human p53 protein.”

p-p53 Cat#9284/RRID: AB_331464
Antibody was validated against human by the manufacturer, stating “Phospho-p53 (Ser15) Antibody detects endogenous levels of p53 only when phosphorylated at serine 15. The antibody does not cross-react with p53 phosphorylated at other sites.”

p21 Cat#2947/ RRID:AB_823586
Antibody was validated against human by the manufacturer, stating “p21 Waf1/Cip1 (12D1) Rabbit mAb detects endogenous levels of total p21 protein. The antibody does not cross-react with other CDK inhibitors.”

phospho-CHK1 Cat#2348/RRID:AB_331212
Antibody was validated against human by the manufacturer, stating “Phospho-Chk1 (Ser345) (133D3) Rabbit mAb detects endogenous levels of Chk1 only when phosphorylated at serine 345.”

phospho-CHK2 Cat# 2661/RRID:AB_331479
Antibody was validated against human by the manufacturer, stating “Phospho-Chk2 (Thr68) Antibody detects endogenous levels of Chk2 only when phosphorylated at threonine 68. The antibody does not cross-react with Chk2 phosphorylated at other sites.”

phospho-histone H2A.X Cat#9718/RRID: AB_2118009
Antibody was validated against human by the manufacturer, stating “Phospho-Histone H2A.X (Ser139) (20E3) Rabbit mAb detects endogenous levels of H2A.X only when phosphorylated at Ser139.”

alpha-Tubulin Cat#2144/RRID:AB_2210548
Antibody was validated against human by the manufacturer, stating “The α-Tubulin Antibody detects endogenous levels of total α-tubulin protein, and does not cross-react with recombinant β-tubulin.”
Antibodies from Santa Cruz Biotechnology:
Vinculin, Cat#sc-73614/ RRID: AB_1131294
Antibody was reported to detect human vinculin by the manufacturer, stating “vinculin (7F9) is a mouse monoclonal antibody raised against vinculin of human origin. Vinculin Antibody (7F9) is recommended to detect vinculin of human, mouse, rat and avian origin”

beta-actin Cat#sc-47778/RRID:AB_626632
Antibody was reported to detect human vinculin by the manufacturer, stating “β-Actin (C4) is a mouse monoclonal antibody raised against gizzard Actin of chicken origin. beta-Actin Antibody (C4) is recommended to detect β-Actin of mouse, rat, human, avian, bovine, canine, porcine, rabbit, Dictyostelium discoideum y Physarum polycephalum origin

Antibodies from Atlas Antibodies:

GCLM Cat# HPA023696/RRID:AB_1849560
Antibody was verified to detect human GCLM by the manufacturer, stating “Orthogonal validation of protein expression using WB by comparison to RNA-seq data of corresponding target in high and low expression cell lines”

Antibodies from Abcam:
Anti- DDDDK tag (FLAG) from Abcam (ab49763/RRID:AB_869428)
Antibody was verified to detect DDDDK tag (FLAG) in a species independent manner. This antibody was stated to detect DDDDK - tagged proteins by Western Blot by the manufacturer, stating that “This is a useful tool for the localisation and characterisation of DDDDK tagged proteins (Binds to FLAG® tag sequence”

BD Biosciences antibodies:
BD Biosciences Cat# 559763, RRID:AB_2869265 - Annexin V
Antibody is routinely tested by flow cytometry by the manufacturer. No information about validation is provided.

Other antibodies:
Antibody against ADH5 was obtained from KJ Patel laboratory (Pontel, et al 2015). We confirmed the validation by using extracts from Nalm6 cells deficient in ADH5.

Eukaryotic cell lines

Policy information about cell lines

Cell line source(s)	HCT116 and HCT116 ΔTP53 were obtained from G. Soria´s laboratory, originally generated by Vogelstein laboratory (DOI: 10.1126/science.282.5393.1497). Nalm6 and DT40 were from KJ Patel laboratory (DOI: 10.1038/nature23481; and DOI: 10.1038/nature10192). ΔFANCB Nalm6 was originally from H. Koyama (DOI: 10.1111/j.1365-2443.2007.01124.x), and ΔADH5 Nalm6 was generated in DOI: 10.1038/nature23481. HAP1 was from KJ Patel laboratory, originally from Haplogen. HepG2 was from KJ Patel laboratory, originally from ATCC collection. HEK293T was a gift from M. Rossi laboratory, originally from ATCC collection. PD20 cells were obtained from V. Gottifredi laboratory, originally from Coriell repositories
Authentication	None of the cell lines used were authenticated. Cell lines generated in this study by CRISPR were validated by western blot and sequencing as described in Methods section.
Mycoplasma contamination	Cell lines were periodically tested for Mycoplasma contamination, testing negative.
Commonly misidentified lines (See ICLAC register)	None of the cell lines used in this study is listed as Misidentified Cell Line in ICLAC v10

Animals and other organisms

Policy information about studies involving animals; ARRIVE guidelines recommended for reporting animal research

Laboratory animals	Caenorhabditis elegans strains obtained from Caenorhabditis Genetics Center (CGC): wild type (N2); JV1, unc-119(ed3);jrIs1 [rpl-17p::HyPer + unc-119(+)]; CL2166, dvIs19[(pAF15)gst-4p::GFP::NLS]<br>We produced the C. elegans strains in the Schumacher Lab (Björn Schumacher, BJS): BJS886, adh-5(sbj21); BJS1006, adh-5(sbj21);Ex [padh-5ADH-5::GFP]<br>We used hermaphrodites in all experiments.
Wild animals	No wild animals were used in the study.
Field-collected samples	No field collected samples were used in the study.
Ethics oversight	This study did not require an ethical approval

Note that full information on the approval of the study protocol must also be provided in the manuscript.

Flow Cytometry

Plots

Confirm that:

- [x] The axis labels state the marker and fluorochrome used (e.g. CD4-FITC).
- [x] The axis scales are clearly visible. Include numbers along axes only for bottom left plot of group (a 'group' is an analysis of identical markers).
- [x] All plots are contour plots with outliers or pseudocolor plots.
- [x] A numerical value for number of cells or percentage (with statistics) is provided.

Methodology

Sample preparation	In the H2DFCDA, PBSF and DHE assays, samples were prepared by lifting cells from 6 or 24-well plates, stained for 30 minutes, washed with PBS and run. For the cell cycle assay, cells were harvested by trypsinization and pelleted by centrifugation (5 min, 1000 x g). Cells were washed with cold PBS and then fixed with 70 % cold ethanol for 15 min on ice. Cells were washed twice with PBS and treated with 30 µg ribonuclease A and 15 µg of propidium iodide. For apoptosis determination, treated cells were harvested, washed with cold PBS and stained with PE-Annexin V antibody and 7-AAD. They were maintained in ice until running on the flow cytometer. For ROS/GSH determination using genetically-encoded sensor, cells were harvested, washed and run on the cytometer.
Instrument	Becton Dickinson's FACS Canto II Flow cytometer and Becton Dickinson's FACS Aria II flow cytometer
Software	BD FACSDivaTM software v6.1 and FlowJo 10.0.7 (Tree Star).
Cell population abundance	> 5000 cells were recorded for the desired population.
Gating strategy	Unless otherwise stated, Initial FSC-A+ and SSC-A+ population was gated using FSC-A and FSC-H to select single cells. This population was used for analysis. The gating approaches are depicted in Supp. Fig 1 and Supp. Fig 3.

- [x] Tick this box to confirm that a figure exemplifying the gating strategy is provided in the Supplementary Information.
